# Breast Cancer Histopathological Images Segmentation Using Deep Learning

**DOI:** 10.3390/s23177318

**Published:** 2023-08-22

**Authors:** Wafaa Rajaa Drioua, Nacéra Benamrane, Lakhdar Sais

**Affiliations:** 1 Laboratoire SIMPA, Département d’Informatique, Université des Sciences et de la Technologie d’Oran Mohamed Boudiaf (USTO-MB), Oran 31000, Algeria; nacera.benamrane@univ-usto.dz; 2 Centre de Recherche en Informatique de Lens, CRIL, CNRS, Université d’Artois, 62307 Lens, France; sais@cril.univ-artois.fr

**Keywords:** semantic segmentation, histopathology, breast cancer, U-Net, convolutional autoencoder

## Abstract

Hospitals generate a significant amount of medical data every day, which constitute a very rich database for research. Today, this database is still not exploitable because to make its valorization possible, the images require an annotation which remains a costly and difficult task. Thus, the use of an unsupervised segmentation method could facilitate the process. In this article, we propose two approaches for the semantic segmentation of breast cancer histopathology images. On the one hand, an autoencoder architecture for unsupervised segmentation is proposed, and on the other hand, an improvement U-Net architecture for supervised segmentation is proposed. We evaluate these models on a public dataset of histological images of breast cancer. In addition, the performance of our segmentation methods is measured using several evaluation metrics such as accuracy, recall, precision and F1 score. The results are competitive with those of other modern methods.

## 1. Introduction

Breast cancer is currently the most common cancer in women. Breast cancer is also the principal cause of death due to cancer in women [1]. Breast cancer tops the list of prevalent cancer types prevalent in Algeria, with more than 14,000 new cases recorded each year (https://www.aps.dz/sante-science-technologie/128390-cancer-en-algerie-65-000-nouveaux-cas-depuis-debut-2021, accessed on 1 December 2020). The best chances of curing are based on an early diagnosis, which will in turn help to detect cancer, allowing for treatment that is generally more effective, less complex and less expensive.

Medical imaging has made significant progress in recent years. The examinations used to detect breast cancer are mammography [2,3], ultrasound [4] and Magnetic Resonance Imaging (MRI) [5]. In the case of the presence of a doubtful or suspicious lesion of cancer, either a nodule [6] or a microcalcification [7], a biopsy is recommended. This biopsy confirms the cancer diagnosis, type and stage [8].

The digitization of tissue samples obtained by taking a sample makes it possible to convert microscopic slides into histopathological images. The automated segmentation of cells from histopathological images of breast cancer is a crucial step for the analysis of cell morphology, which is essential for the diagnosis of different pathologies particularly in oncology [9].

Breast cancer diagnosis has evolved through multiple research techniques over the years, such as segmentation, detection, and classification. The role of detection is to separate and identify the different regions of the image. The most frequently used object detection models are Faster R-CNN (convolutional neural network) [10], Mask R-CNN [11], and YOLO (You Only Look Once) [12].

In the literature, a number of supervised research works have been conducted that apply morphological operations for the segmentation of histopathological images [13]. Various hybridized models have been presented in the literature [14,15]. Qu et al. proposed a pixel-wise classifier Support Vector Machine (SVM)-based method for tumor matrix segmentation and a marker-driven watershed-based method for nuclei segmentation [14]. Rashmi et al. developed a segmentation technique combining a multilayer perceptron (MLP) and SVM for the segmentation of cell images of breast cancer [15]. Abdolhoseini et al. [16] developed a segmentation technique combining multilevel thresholding and the watershed algorithm to separate clustered nuclei.

Faridi et al. [17] presented an automated system for detecting and segmenting cancer cell nuclei that is partially different from the system for segmenting healthy cell nuclei using the level-set algorithm. Jian et al. [18] utilized a thresholding technique using OTSU. Therefore, artificial intelligence-based applications, especially deep convolutional neural network, were used [19].

However, some works on the segmentation of histopathological images have been proposed. Sahasrabudhe et al. [20] proposed a supervised approach for the segmentation of nuclei without annotations. They used a fully convolutional attention network based on advanced filters to generate segmentation maps for nuclei in the image space. Kate et al. [21] developed a model that is based on the particle swarm optimizer (PSO) for the segmentation of breast cancer histopathology images. Shu et al. [22] presented a method to segment highly clustered overlapping cores. The proposed method uses a combined global and local thresholding method to extract foreground regions. Xu et al. [23] proposed an unsupervised method, termed the tissue cluster level graph cut, for segmenting histological images into meaningful compartments (tumor or non-tumor regions). This approach has been evaluated on histological image sets for necrosis and melanoma detection. Khan et al. [24] proposed a framework for unsupervised tumor segmentation based on stromal organization, which was divided into two types: hypocellular stroma and hypercellular stroma. Evaluation of the algorithm was performed using H&E-stained breast histology images. Fouad et al. [25] presented an alternative data-independent framework based on the unsupervised segmentation of oropharyngeal cancer tissue micro-arrays from histological images.

Following the great success of convolutional neural networks in image analysis, we used deep learning architectures to study the problem of nuclei segmentation in breast cancer histopathological images. Firstly, we presented an unsupervised architecture using an autoencoder to avoid manually calculating the characteristics of the k-means clustering input. Secondly, we developed an improved U-Net for semantic segmentation.

The paper is organized as follows. Section 2 presents related work. Section 3 describes the methodology and the different architectures used in this work. Section 4 presents the results and discussion. The comparative study is described in Section 5, which is followed by the conclusion and some futures perspectives in Section 6.

## 2. Related Works

In recent years, deep learning CNNs have dominated many areas of computer vision applications. The section will review the state of the art of CNN-based methods for nuclei segmentation from histopathological images.

Chan et al. [26] proposed a method for the semantic segmentation of histological tissue (HistoSegNet). The authors trained a convolutional neural network on patch annotations and inferred gradient-weighted class activation maps with average overlapping pre- dictions. Cui et al. [27] introduced a nucleus-boundary model, which used a fully convolutional neural network to simultaneously predict the nucleus and its boundaries. The experimental results show that the proposed method outperformed prior state-of-the-art methods. Paramanandam et al. [28] proposed a segmentation algorithm for detecting single nuclei from breast histopathology images stained with hematoxylin and eosin. The recognizer estimates a nuclei saliency map using boundary extraction using a Loopy Back Propagation (LBP) algorithm on a Markov random field. Naylor et al. [29] formulated the segmentation problem as a distance map regression problem. The authors demonstrate a performance of the method compared to other methods using CNN. Veta et al. [30] described the results from the Assessment of Mitosis Detection Algorithms 2013 (AMIDA13). Zemouri et al. [31] proposed a breast cancer computer-aided diagnosis based on constructive deep neural network and joint variable selection. This contribution outperformed the use of the deep learning architecture alone. Jafarbiglo et al. [32] presented an automatic diagnostic system that classifies histopathological images based on the nuclear atypia criterion using a CNN-based method. Kang et al. [33] applied four parallel backbone nets, which were merged by the attention generation model. Kaushal et al. [34] recently summarized techniques for breast cancer diagnosis using histopathological images. Wahab et al. [35] used the concept of transfer learning by first using a pre-trained convolutional neural network for segmentation and then another hybrid-CNN for the classification of mitoses.

Qu et al. [36] used the fully connected conditional random field loss for further refinement. The model did not introduce extra computational complexity during inference. Xu et al. [37] presented a deep convolutional neural network (DCNN)-based feature learning to automatically segment or classify epithelial and stromal regions from digitized tumor tissue microarrays and then compared DCNN-based models with three handcraft feature extraction-based approaches. Sohail et al. [38] proposed a CNN-based deep multiphase mitosis detection framework for identifying mitotic nuclei in breast cancer histopathology images. The authors developed an automatic label refiner to render weak labels with semantic information for the purpose of training deep CNN. Cao et al. [39] presented an automated method for breast cancer scoring in histopathology images based on computer-extracted pixel, object, and semantic-level features derived from CNN.

Ozturk et al. [40] proposed an automatic semantic segmentation based on cell type using the structure of novel deep convolutional networks (DCNNs). The authors presented semantic information on four classes, including white areas in the whole-slide image, tissue without cells, tissue with normal cells and tissue with cancerous cells. Kaushal et al. [41] compared various state-of-the-art segmentation techniques for extracting cancer cells in histopathology images using the triple-negative breast cancer dataset. Gour et al. [42] developed a deep residual neural network model (DeepRNNetSeg) for automatic nucleus segmentation on histopathological breast cancer images.

Deep learning approaches, in particular via auto-encoding architectures, make it possible to avoid manually defining the characteristics by computing a compressed representation of an image in a latent space via applying convolutional filters. Most of the work has been used to perform segmentation or kernel detection. Xu et al. [43] presented a stacked sparse autoencoder for the efficient detection of nuclei from high-resolution histopathological images of breast cancer. Raza et al. [44] summarized various unsupervised deep learning models, tools, and benchmark datasets applied to medical image analysis. Janowczyk et al. [45] presented stain normalization using sparse autoencoders (StaNoSA) to normalize the color distribution of test image. The architecture was applied on digital histopathology slides. Hou et al. [46] developed an unsupervised detection network by exploiting the properties of histopathological images. They identified nuclei in image patches in tissue images and encoded them into a feature map encoding the location of the nuclei.

## 3. Methodology

The first method is designed for the unsupervised segmentation of overlapping nuclei, and the second method aims to segment regions of the nuclei. In this section, we present our two methods.

### 3.1. Segmentation-Based Deep Learning Cluster Architecture

**Figure 1:**
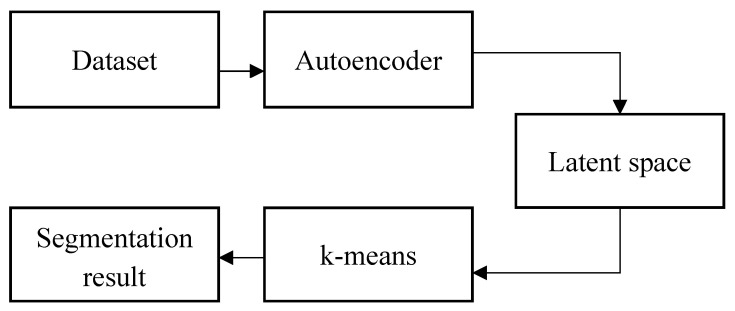
Steps of unsupervised segmentation method.

Our framework for learning the neural network parameters and cluster assignments is based on a deep learning cluster architecture, as shown in Figure 1.

**Figure 2:**
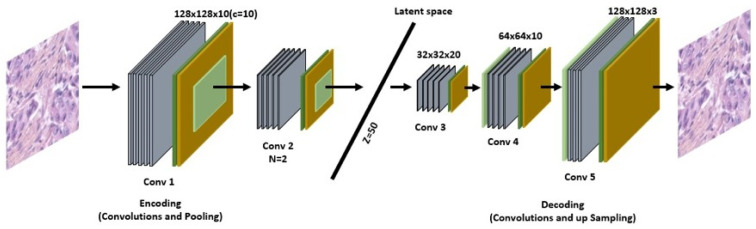
Proposed approach for unsupervised segmentation.

**Figure 3:**
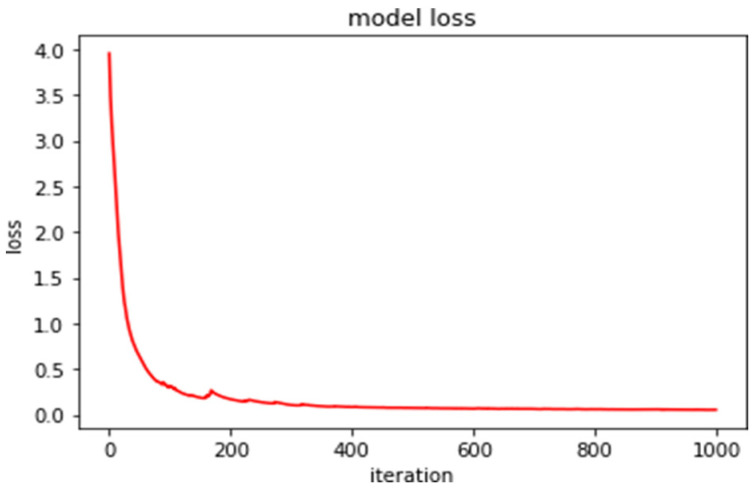
Proposed approach for unsupervised segmentation.

In this section, we investigate the potential of using convolutional autoencoders for clustering histopathological images. As shown in Figure 2, a convolutional autoencoder (CAE) is a deep convolutional neural network consisting of two parts: an encoder and a decoder. The main purpose of CAE is to minimize a reconstruction loss, a function evaluating the difference between the input and the output of the CAE, as shown in Figure 3. Once this function is minimized, it can be assumed that the encoder part establishes a proper abstract of the input data in latent space, because the decoder part is able to reconstruct a strongly similar copy of it from this encoded representation. The CAE is described in Algorithm 1.
Algorithm 1Input: Image set X = {x}, Network Net (N, C, Z)Initialize the network parameters N, C, ZRepeatUpdate network parameters by minimizing reconstruction loss until convergence For each image x in X, doGenerate reconstruction image xR from Net (x, N, C, Z)End for Output: reconstruction image xR representation in latent space Z

Figure 2 shows our proposed network architecture. The autoencoder is used for accurate image segmentation. We trained this autoencoder using the encoder’s weights and added another branch for clustering.

The encoder consists of an input layer (of the size of the input image), which is connected to N convolution layers of decreasing size, up to an information bottleneck of size Z, which is called latent space. The latent space is connected to a series of layers of N convolutions of increasing size until reaching the size of the input image. This second part is called the decoder. Each convolution layer is composed of C convolutions and is followed by three other layers: a normalization, an activation function (ReLU), and a max pooling of size (2,2).

The first step is to train an autoencoder using a set of unlabeled images. An autoencoder consists of an encoder network and a decoder network. The encoder compresses the input image into a lower-dimensional latent representation, while the decoder reconstructs the image from the latent representation. The autoencoder is trained to minimize the difference between the original input and the reconstructed output, effectively learning to capture meaningful features in the data.

Once the autoencoder is trained, the encoder network is used to extract the latent space representation of each image in the dataset. These latent representations capture the essential characteristics of the images in a compact form.

To perform clustering, a trained CAE is used to encode each part of the image. Then, the coded representation in the latent space is given as input to a K-means clustering algorithm, which assigns it a cluster. One of the main challenges of unsupervised clustering is to find the correspondence between a cluster and a class. In our case, the problem is expressed as a two-class problem: tumor or no-tumor.

The next step is to apply the K-means clustering algorithm to the extracted latent representations. K-means clustering is an iterative algorithm that aims to partition data points into two clusters. The algorithm finds the cluster centers that minimize the sum of squared distances between the data points and their respective cluster centers. In this case, the latent representations serve as the input data for the K-means algorithm.

Once the K-means algorithm has converged, each latent representation is assigned to one of the 2 clusters based on its proximity to the cluster centers. This assignment determines the segmentation labels for each input image.

To perform segmentation on a new image, it is first passed through the trained encoder to obtain its latent space representation. Then, the representation is assigned to one of the 2 clusters using the K-means algorithm. Finally, the corresponding cluster label is assigned to each pixel in the image, resulting in a segmented image.

### 3.2. Segmentation Based on Improved U-Net

A framework is proposed to automatically segment nuclei regions and overlapping nuclei regions.

**Figure 4:**
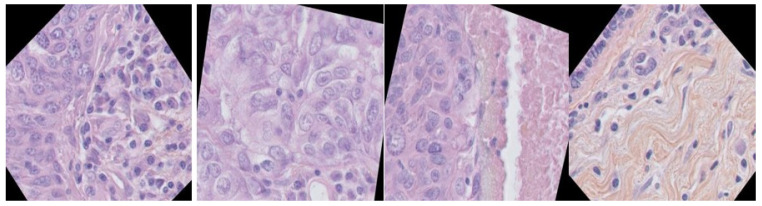
Data augmentation.

In order to train our model, we need a significant amount of data. The quantity and quality of our dataset will play an important role in the development of a good model; the data augmentation will be of great use to us. The principle of data augmentation is based on the principle of artificially augmenting our data by applying transformations. We will be able to increase the diversity and, thus, increase the learning domain of our model. There are several techniques which are most often used, such as rotation, saturation, brightness, and noise. In this work, we used the rotation technique with different angles, as seen in Figure 4; this step was carried out by the Roboflow library [47]. This provided most of the tools needed to convert raw images into a custom-trained computer vision model and deploy them for use in our applications.

In histopathological images, semantic segmentation aims to label each pixel with one of two diagnoses (cancerous/non-cancerous). These methods include slider-based methods that train and predict at the pixel of the slider patch to obtain predictions.

**Figure 5:**
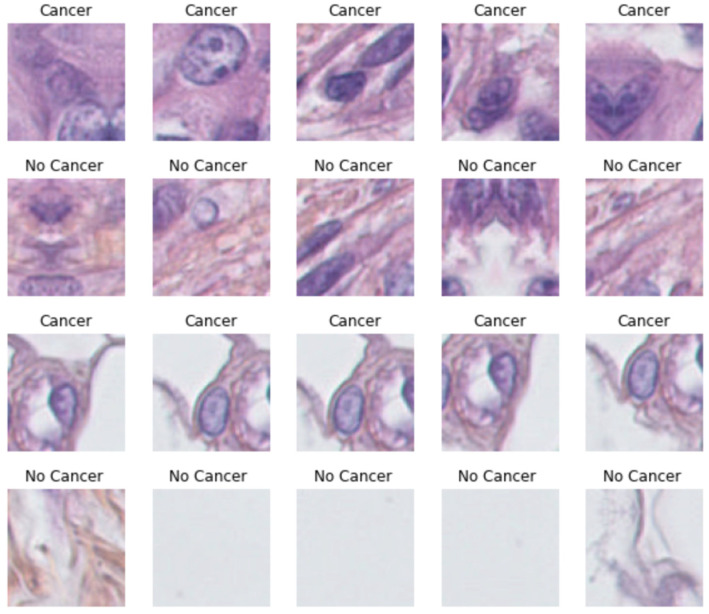
Data preprocessing.

To validate the proposed model, firstly, we prepared the data (data preprocessing). This consisted of dividing each image into patches 32 × 32 in size in the U-Net model. This operation classified our data into two groups, cancer or non-cancer, based on ground truth. Figure 5 illustrates an example of this processing.

The U-Net was proposed by [48] for the segmentation of biomedical images, where training data are often scarce. The encoder network and decoder network form a U-shaped architecture. In the encoder path, convolutional/max pooling layers reduce spatial information while increasing feature information. In the decoder path, feature maps and spatial information are combined with high-resolution features from the decoder path through a series of up-convolutions and concatenations. The proposed method consists of re-designing a U-Net network structure with new layers. To validate the proposed model, firstly, we prepared the data (data preprocessing), and then, the model was trained. As the output, the final vector allowed for a fine-grained prediction of binary classes (tumor or non-tumor).

**Figure 6:**
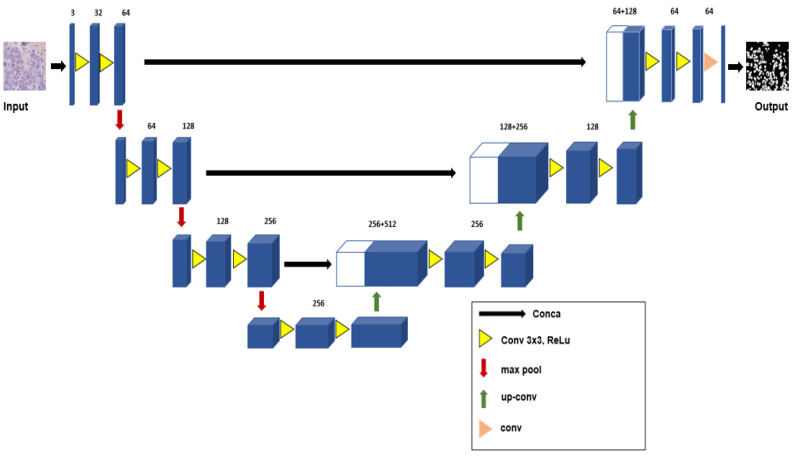
U-Net architecture.

The U-Net network has a deconvolution part symmetrical to the convolution part, which makes it possible to obtain feature maps whose sizes are compatible between the two parts of the network. Thus, the feature maps extracted in the convolution part could be concatenated to those reconstructed in the deconvolution part, thus transforming more important spatial information and allowing for better reconstruction. The addition of new layers in the encoder and decoder parts (as seen in Figure 6) allows for better collaboration between the different feature maps and improves the recognition capacity of the network.

Encoder: The encoder part of a U-Net typically consists of convolutional layers, max pooling, and non-linear activation functions. The number of these layers can suit specific requirements by, for example, increasing the depth or changing the filter sizes.

Decoder: The decoder part of U-Net performs up-sampling and concatenates the corresponding encoder features with the up-sampled features. The goal is to recover the spatial resolution lost during the encoding process. We can modify the decoder architecture by changing the up-sampling method, adjusting the number and type of layers, or using skip connections for better feature fusion.

Output layer: The U-Net typically uses a 1 × 1 convolutional layer with a softmax activation function to generate the final pixel-wise segmentation output.

Skip connections: U-Net uses skip connections to propagate information from the encoder to the decoder, aiding in better feature fusion.

Loss function: An appropriate loss function is selected for semantic segmentation. A commonly used loss function is pixel-wise softmax loss. The choice of loss function depends on the specific characteristics of the dataset.

(1) $$p_{k}\left(x\right)=\exp\left(a_{k}\left(X\right)\right)/\left(\sum_{k^{\prime }=1}^{k}exp\left(a_{k^{\prime }}(X)\right)\right)$$

where p_k_(x) denotes the activation in feature channel k at the pixel position x.

**Figure 7:**
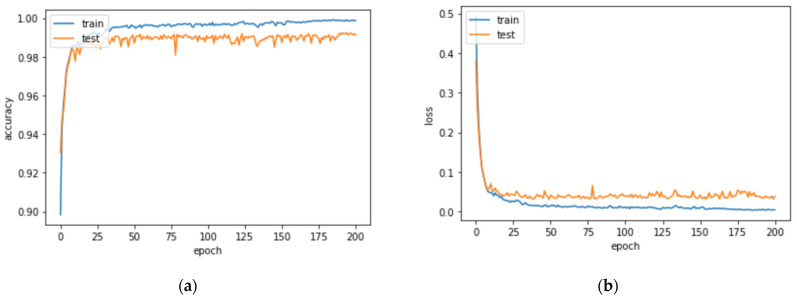
Performance of the U-Net network on training datasets: (**a**) plot of loss, (**b**) plot of accuracy over the training epoch. U-Net architecture.

The models used in our work were trained over 200 epochs using a TensorFlow framework in an environment with GPU (Nvidia 1080-ti), 16 GB of RAM, and CPU-i7 intel. The figures illustrate the performance of the network on training datasets. Figure 7 shows the plot of loss and accuracy over the training epoch of U-Net.

## 4. Results and Discussion

**Figure 8:**
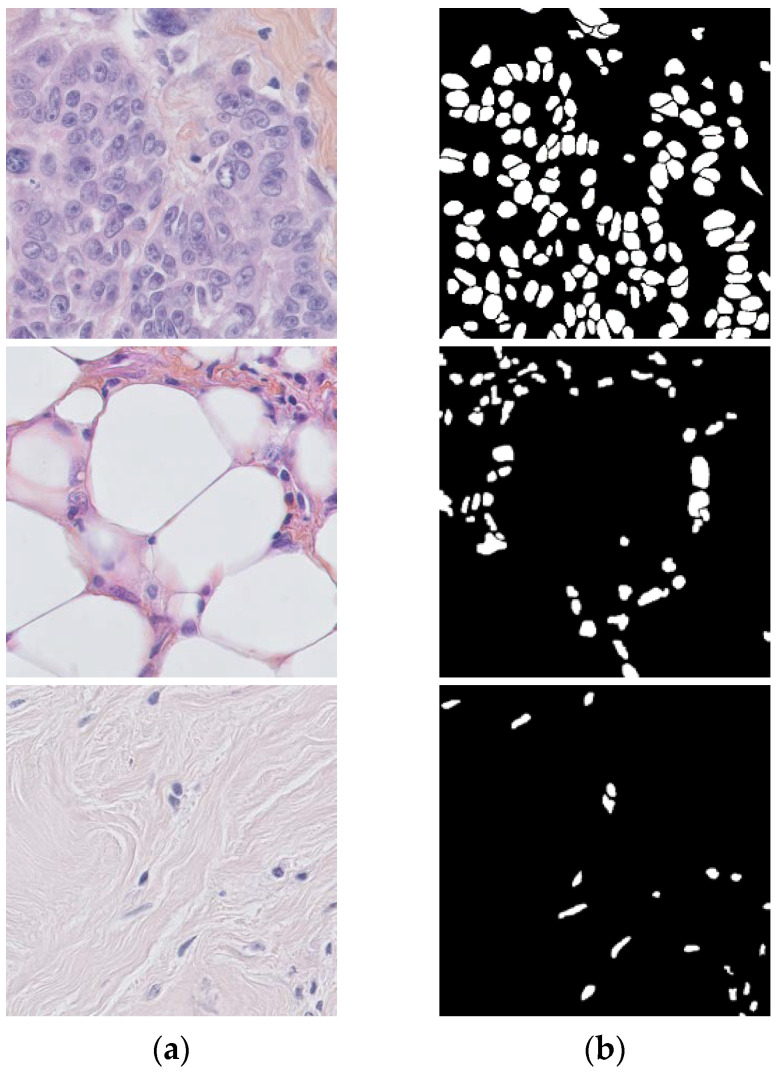
Example of datasets, (**a**) the original data, (**b**) the ground truth.

The data used in this work were from the breast cancer histopathology image dataset (BNS), which was introduced in [49]. The annotated dataset provides images grouped by patient. Each patient has histopathology data annotated with their ground truth. The size of this dataset 512 × 512 pixels label data; see Figure 8 for this dataset. This study analyzed publicly available datasets. These data can be found here: https://github.com/wafaadrioua/Hystopathological (accessed on 17 August 2023).

### Experimental Tests

The results for each approach are given in Table 1, which contains the results of the segmentation methods as well as the results of the FCN on the same data, which was carried out as a reference base in order to assess the relevance of the improved U-Net network on one side and the data encoding on the other side (CAE).

The FCN (fully convolutional network) was proposed by [50], which adopts a pre-trained CNN for image classification as the encoder module of the network. A fully connected layer was connected to a convolutional layer by reusing its weights and biases. A decoder module with transposed convolutional layers was added to upscale feature maps to obtain full-resolution segmentation maps. Here, AlexNet is the basic network of the FCN model.

First of all, we note that the FCN applied to the data obtained results of lower quality than the two proposed approaches, which confirms that the addition of a layer in the U-Net network as well as the clustering from the encoded data have a positive effect on segmentation.

The main objective of this study was to design new models within the framework of histopathological image segmentation based on three CNN architectures.

**Figure 9:**
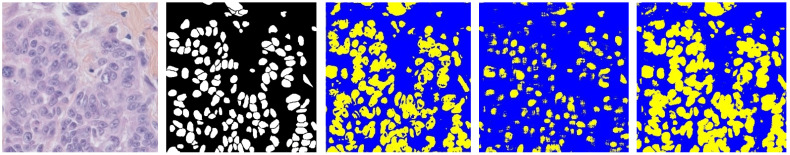

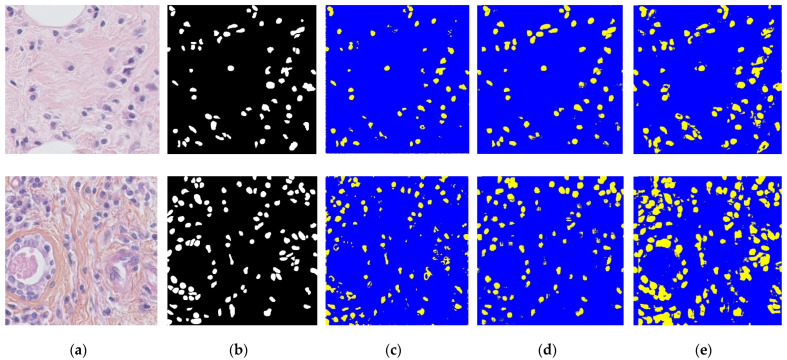
The obtained results. (**a**) The original data, (**b**) the ground truth, (**c**) the U-Net results, (**d**) the deep clustering results, (**e**) the FCN results.

Figure 9 shows three visual examples of breast cancer histological images. In Figure 9, row (a) presents original images from the dataset, row (b) presents ground truth segmentations, (c) presents the results obtained for proposed approach 1, (d) presents the results obtained for the proposed approach, and (e) presents the obtained FCN results. Rows 3 and 4 compare tumor and non-tumor cells using two techniques, where yellow indicates automatically segmented tumor cell regions and blue indicates a background.

For the comparison experiments, different metrics were used such as precision, recall, accuracy, F1 score and IoU (intersection over union) [51] to evaluate the proposed models.

(2) $$Precision=\frac{TP}{TP+FP}$$

(3) $$Recall=\frac{TP}{TP+FN}$$

(4) $$F1=2*\frac{Recall*Precision}{Recall+Precision}$$

(5) $$Accuracy=\frac{TP+TN}{TP+FP+TN+FN}$$

(6) $$IoU=\frac{TP}{TP+FP+FN}$$

where TP, FP, FN, and TN are the true positive, false positive, false negative, and the true negative, respectively.

As can be observed in Table 1, the proposed U-Net model trained with manual annotations and an unsupervised segmentation provided a comparative performance on the dataset, with IoU values of 86.1% and 84.8%, respectively.

From these evaluations, it can be concluded that the proposed unsupervised method can provide a comparable cell segmentation performance compared to the modified U-Net model. Furthermore, as collecting manual histological annotations is time-consuming and expensive, unattended methods can potentially be used to create histological annotations in order to train supervised segmentation methods.

## 5. Comparison Study with the Existing Works

We conducted comparative studies with other works in the literature. We selected some works from the deep learning-based literature (as seen in Table 2). The experimental results show the efficiency of our proposed architecture compared to other works.

Our method outperformed the current state-of-the-art methods using the dataset (described in the dataset section) in terms of completeness and segmentation accuracy for single-nuclei segmentation, especially when segmenting overlapping regions of the nuclei. We compared our method with several deep learning-based methods listed in Table 1, such as the modified U-Net and unsupervised model.

## 6. Conclusions

This paper presents a novel unsupervised technique for histological image segmentation. The proposed unsupervised approach segments nuclei into two clusters based on an autoencoder model. Both autoencoders and U-Net have shown promising results in histopathology image segmentation. Autoencoders have the advantage of unsupervised learning, allowing them to leverage unlabeled data for representation learning. On the other hand, the improved U-Net is a supervised approach that requires labeled training data but offers a more specialized architecture for segmentation tasks.

The experimental results evaluated on histopathology image sets with different color staining methods show that the unsupervised method can effectively segment the tested histological image into tumor or non-tumor cells. In particular, it provides a comparative segmentation performance with a proposed U-Net model in terms of nuclei segmentation. Unattended methods are a general image segmentation framework that can be extended to solve various image segmentation problems. Furthermore, due to their unsupervised nature, when histological annotations are difficult to collect, unsupervised methods can be used to generate image annotations to train supervised segmentation models such as U-Net. The results of our work show that our models are more robust in comparison with the methods in the literature. In the future, we will explore more advanced methods with state-of-the-art segmentation techniques.

## Figures and Tables

**Table 1:** Metrics results.

Models		Accuracy	Recall	Precision	F1 Score	IoU
U-Net proposed	Image1	0.986	0.911	0.896	0.909	0.861
	Image2	0.891	0.870	0.811	0.910	0.857
	Image3	0.809	0.899	0.840	0.902	0.834
Unsupervised approach	Image1	0.857	0.902	0.955	0.893	0.807
	Image2	0.860	0.875	0.866	0.907	0.848
	Image3	0.822	0.823	0.853	0.834	0.810
FCN	Image1	0.805	0.891	0.855	0.823	0.723
	Image2	0.855	0.838	0.806	0.893	0.803
	Image3	0.819	0.861	0.825	0.823	0.792

**Table 2:** Metrics results.

Approaches	Accuracy	Recall	Precision	F1 Score	IoU
PangNet [49]	0.924	0.665	0.814	0.676	0.722
DeconvNet [49]	0.954	0.773	0.864	0.805	0.814
Ensemble [49]	0.944	0.900	0.741	0.802	0.804
DCNN/U-Net [52]	0.94	0.60	0.90	0.70	0.55
NucSeg-N [53]	N/A	0.910	**0.910**	**0.909**	N/A
NucSeg-P [53]	N/A	0.886	0.893	0.887	N/A
NucSeg-NP [53]	N/A	0.889	0.912	0.899	N/A
Unsupervised approach	0.860	0.875	0.866	0.907	0.848
U-Net proposed	**0.986**	**0.911**	0.896	**0.909**	**0.861**
FCN	0.819	0.861	0.825	0.823	0.792

## Data Availability

Dataset is available online at GitHub: https://github.com/wafaadrioua/Hystopathological (accessed on 17 August 2023).
